# Distribution of Diverse *Escherichia coli* between Cattle and Pasture

**DOI:** 10.1264/jsme2.ME17030

**Published:** 2017-09-27

**Authors:** Gitanjali NandaKafle, Tarren Seale, Toby Flint, Madhav Nepal, Stephanus N. Venter, Volker S. Brözel

**Affiliations:** 1 Department of Biology and Microbiology, South Dakota State University Brookings, South Dakota United States of America; 2 Department of Microbiology and Plant Pathology, University of Pretoria Pretoria South Africa; 3 Department of Mathematics and Statistics, South Dakota State University Brookings, South Dakota United States of America

**Keywords:** *Escherichia coli*, niche partitioning, pasture, bovine feces, cattle, phylogroup

## Abstract

*Escherichia coli* is widely considered to not survive for extended periods outside the intestines of warm-blooded animals; however, recent studies demonstrated that *E. coli* strains maintain populations in soil and water without any known fecal contamination. The objective of this study was to investigate whether the niche partitioning of *E. coli* occurs between cattle and their pasture. We attempted to clarify whether *E. coli* from bovine feces differs phenotypically and genotypically from isolates maintaining a population in pasture soil over winter. Soil, bovine fecal, and run-off samples were collected before and after the introduction of cattle to the pasture. Isolates (363) were genotyped by *uidA* and *mutS* sequences and phylogrouping, and evaluated for curli formation (Rough, Dry, And Red, or RDAR). Three types of clusters emerged, *viz.* bovine-associated, clusters devoid of cattle isolates and representing isolates endemic to the pasture environment, and clusters with both. All isolates clustered with strains of *E. coli sensu stricto*, distinct from the cryptic species Clades I, III, IV, and V. Pasture soil endemic and bovine fecal populations had very different phylogroup distributions, indicating niche partitioning. The soil endemic population was largely comprised of phylogroup B1 and had a higher average RDAR score than other isolates. These results indicate the existence of environmental *E. coli* strains that are phylogenetically distinct from bovine fecal isolates, and that have the ability to maintain populations in the soil environment.

*Escherichia coli* occurs as part of the intestinal microbiota of many warm-blooded animals, their primary habitat, and were considered to survive for only short periods outside the host (47). The use of *E. coli* as an indicator organism is based on the assumption that it does not persist and grow in secondary environments such as soil, water, and sediments, thereby indicating the presence of recent fecal contamination (33, 42). Recent studies demonstrated that *E. coli* has the ability to maintain populations in aquatic and soil environments (29, 58). The occurrence of *E. coli* in soil, sediments, and water in tropical and sub-tropical regions has been widely documented, and the species is now considered to be autochthonous to soil within such warm regions (9, 10, 13, 17, 23, 48). *E. coli* may also survive for long periods and potentially replicate in temperate environments. Strains have been repeatedly isolated from the undisturbed riparian soils of Southern Lake Michigan, Indiana (12). Ishii *et al.* (27) also reported the isolation of naturalized *E. coli* strains from the temperate soil of Lake Superior Watershed, Minnesota. *E. coli* were also found attached to the macro-alga *Cladophora* in Lake Michigan (11), to periphyton in Lake Superior (32), and in beach sand and sediments (4, 28). Persistent strains have also been reported from alpine pasture soils, whether sampled from under or away from cowpats (50).

The genus *Escherichia* comprises *E. coli*, *E. hermanii*, *E. vulneris*, *E. fergusonii*, and *E. albertii*, with *E. marmotae* recently being added (38). Only *E. coli* contains a functional β-glucuronidase encoded by *uidA* (24), allowing its distinction on differential media such as Membrane Lactose Glucuronide Agar (MLGA). Although enterohemorrhagic *E. coli* O157:H7 lacks functional β-glucuronidase, positive variants have been reported (24, 46). A collection of environmental *Escherichia* isolates initially considered to be *E. coli* have been assigned to four distinct genetic clusters based on their unique Multi-Locus Sequence Type profiles, and named clades I, III, IV, and V (54). According to the extent of recombination between isolates of *E. coli*, Clade I is viewed as part of *E. coli sensu stricto* (39), whereas clades III, IV, and V are phylogenetically distant and not part of that species. Environmental *E. coli* may be defined as resident in a primary habitat in an environment outside of the host. Clade I is the most closely related to *E. coli*, with evidence for genetic exchange between members of the two. Clade strains are phenotypically similar to *E. coli*, and generally positive for β-glucuronidase; however, clade III cannot ferment sorbitol and sucrose or utilize lysine (54). They may be distinguished from *E. coli sensu stricto* by multi-locus sequence typing (MLST) and phylogrouping.

There is increasing evidence to show that *E. coli* strains occurring in secondary environments are genetically and phenotypically distinct from *E. coli* inhabiting the gut (21). The allocation of *E. coli* to four phylogroups by multiplex PCR (15) has indicated a degree of niche partitioning. Phylogroups A and B1 appear to be dominated by environmentally occurring strains (53), whereas B2 and D are predominated by mammalian isolates (18, 35). These findings suggest the niche partitioning of diverse *E. coli* strains across various environments, and, thus, some strains may be autochthonous to soils. This phylogrouping protocol was recently refined to yield seven groups (16). Isolates from the temperate soil of Lake Superior Watershed displayed DNA fingerprints distinct from animal-derived isolates (28). *E. coli* from fresh water beaches along Lake Huron and the St. Clair River in Michigan revealed the extensive genetic diversity of MLST (53). The *uidA* sequences of *E. coli* from alpine pasture soil were distinct from fecal *E. coli*, indicating a naturalized population that was part of the indigenous soil community (50).

Previous studies have established the occurrence of *E. coli* in soil, water, and sediments under various climatic conditions. The objective of the present study was to investigate whether niche partitioning of *E. coli* occurs between cattle and their pasture. We attempted to clarify whether *E. coli* from bovine feces differ phenotypically and genotypically from isolates maintaining a population in pasture soil over winter. *E. coli* strains that survived in pasture soil through the extreme South Dakota winter displayed a different genotype from bovine fecal isolates, and need to be considered as environmental or naturalized.

## Materials and Methods

### Sample collection

Samples were collected from a cattle pasture (12.14 ha) divided into four separate encampments (GPS co-ordinates 44°22′17.70″N 96°58′1.54″W) at Volga, SD, USA in May, June, and July, 2013. This pasture had been cleared of cattle at the end of July 2012. Before the reintroduction of five or more cows per encampment, soil cores (4 cm in depth) were collected over three weeks, between May and June, and designated Soil Before Grazing (SBG). Following the introduction of cattle at the start of July, soil cores, run-off, and cattle fecal samples were collected once every week for four weeks and from each of the four encampments. Every week, a transect was drawn at random across each encampment, and five soil samples, five run-off samples, and two fresh fecal samples were collected per transect. Soil samples were taken to a depth of 4 cm using a soil borer (2 cm in diameter), and soil cores transferred to sterile 50-mL conical screw-cap tubes. A simulation of run-off was performed using a Cornell infiltrometer (41) fed with sterile dH_2_O. Run-off was collected into sterile 100-mL screw-cap flasks. Fresh fecal samples were taken directly from the pasture by scooping into sterile 50-mL conical screw-cap tubes. Samples were transported to the laboratory in a cooled container and processed on the same day. Run-off and soil samples collected at the time of grazing were designated as pasture samples.

### Isolation of *E. coli* from soil, run-off, and fecal sample

Twenty grams of soil were mixed vigorously with 100 mL sterile water and allowed to settle for 1 h. One- and ten-mL aliquots were then filtered through a sterile 0.45-μm mixed cellulose ester filter (Millipore), which was placed on Membrane Lactose Glucuronide Agar (MLGA, Fluka Analytical). Run-off samples (1 and 10 mL) were filtered directly through 0.45-μm nitrocellulose filters, which were then placed on MLGA. Aliquots of ten-fold dilutions of bovine fecal samples were plated on MLGA. Green colonies were positive for β-Galactosidase and β-Glucuronidase activities, and were assumed to be *E. coli*. This protocol excluded β-glucuronidase-negative O157:H7 strains. An average of 2 colonies were picked from the highest dilutions showing growth, streaked onto MLGA to confirm purity, sub-cultured onto LB agar, and stored at –80°C in 50% glycerol. All 15 cryptic species isolates obtained from Dr. Seth Walk grew on MLGA and 8 formed green colonies; therefore, it is unlikely that our isolation method excluded potential members of clade I, III, IV, or V (data not shown).

### Analysis of *uidA* and *mutS* gene sequences

Genomic DNA was extracted from overnight LB agar cultures suspended in 10 mM phosphate buffer (pH 7.0) using the genomic DNA Quick Prep Kit (Zymo Research), and stored at –20°C. The *uidA* and *mutS* genes were amplified by PCR using primers described previously (54) (Table S1), with *E. coli* MG1655 as a positive control. PCR reactions (25 μL) were set up as follows: 2.5 μL reaction buffer (10X) (New England Biolab), 0.5 μL MgCl_2_ (25 mM), 0.5 μL dNTPs (40 mM), 0.1 μL forward primer and 0.1 μL reverse primer (100 μmol), 0.125 μL of Taq polymerase (NE Biolab), 0.5 μL of a DNA template, and 20.7 μL of sterile nano pure water. The amplification cycle was initiated with 95°C for 2 min, followed by 30 cycles of denaturing at 95°C for 30 s, annealing at 56°C for 30 s, and extension at 72°C for 1 min, with a final extension at 72°C for 5 min. DNA sequences were elucidated by the dideoxy chain termination method (Beckman Coulter Genomic Center at Denver, MA). *uidA* sequences were submitted to GenBank (http://www.ncbi.nlm.nih.gov/genbank/) under the BankIt number 1841773 (accession numbers KT311394–KT311756), and *mutS* sequences as the BankIt number 1841687 (accession numbers KT311004–KT311366). DNA sequences were aligned using ClustalW (49), and overhangs were trimmed using SeAl (Rambaut, A. 2002. http://tree.bio.ed.ac.uk/software/seal/). The *uidA* and *mutS* sequences for all isolates and reference strains (36) were concatenated using SeAl. A maximum likelihood analysis using model GTR+G+I with 1,000 bootstrap replicates was performed in the program MEGA6.06 (49). The tree was then annotated and visualized using the ITOL online tool (37).

### Identification of phylogroups

Isolates were assigned to phylogroups using the protocol described by Clermont *et al.* (16). In order to avoid ambiguity, PCR was performed separately for each primer set (Table S1). In order to clarify whether the distribution of phylogroups differed by source or cluster, we used multinomial log-linear regression models. A multiple logistic regression is used when the dependent variable is nominal and there is more than one independent variable. It is a classification method that generalizes logistic regression to multiclass issues having more than two possible discrete outcomes (40). The models were fit using the nnet package in R (v.3.2.2) (44). The response variable in this analysis was the phylogroup of each isolate (A, B1, B2, C, D, E, and Unknown), and the explanatory variables were the sample source and clusters associated with the origin of the isolates. In order to visualize the effects of significant explanatory variables, we used regression trees fit using Package Party (26) in R.

### Identification of the RDAR (Red, Dry, And Rough) morphotype

The RDAR morphotype was identified as described by White *et al.* (56). Briefly *E. coli* isolates were grown at 37°C overnight on LBns agar (LB without salt), followed by culturing at 37°C overnight in LBns broth with shaking. Spot colonies were prepared by an inoculation of 1 μL of the overnight broth culture onto LBns agar supplemented with 100 μg mL^−1^ Congo red. Colonies were observed under a stereo microscope (Olympus SZX16) after an incubation at 28°C for 72 h. Colony morphologies were assigned to four groups where white smooth colonies were “ws”, red smooth colonies were “rs”, slight rough colonies were “sc”, and highly wrinkled (curli) colonies were “c”.

### Degree of biofilm formation in LB and SESOM media

Regarding the biofilm quantification in LB media, 5 μL of the overnight broth culture was mixed with 195 μL of LB broth in a 96-well plate and incubated for 16 h at 37°C. *Pseudomonas aeruginosa* (PAO) (obtained from Dr Sang-Jin Suh, Auburn University, Alabama, USA) was used as a positive control. Soluble Extractable Soil Organic Matter (SESOM) was prepared for culturing in liquid soil extract using air-dried soil from the cattle pasture as described previously (51). Five microliters of overnight broth culture was mixed with 195 μL of SESOM, harvested by centrifugation, and the pellet re-suspended in 200 μL of SESOM. The staining and quantification of biofilms were performed using the Crystal Violet (CV) assay as described by (43), but with 95% ethanol instead of 30% acetic acid for the solubilization of CV. Each treatment was repeated four times. Isolates were assigned to four groups based on the quantity of the biofilm formed (absorbance at 560 nm). In SESOM, group “0” was <0.01, group “1” was <0.025, group “2” was <0.05, and group “3” was >0.05. In LB, group “0” was <0.025, group “1” was <0.05, group “2” was <0.1, and group “3” was >0.1.

### Long-term survival

In order to study the long-term survival of *E. coli* in soil under environmental conditions, 45 isolates were selected to represent the three sample sources and three cluster types (see Results). A loopful of culture was then washed two times with sterile water and the pellet suspended in 500 μL SESOM. Two hundred microliters of this cell suspension was inoculated into 20 mL of SESOM and incubated at 25°C overnight. The overnight liquid culture was diluted to an optical density of 0.05 at 546 nm (10^6^ CFU mL^−1^) and 1 mL of this diluted cell suspension was used to inoculate 5 g of double autoclaved pasture soil placed in a sterile 50-mL conical tube. A further 2 mL of sterile dH_2_O was added to moisten the soil, which was shaken vigorously for 1 min, and the culturable count measured for time zero. Each soil microcosm was set up three times. Tubes were placed on the soil surface outdoors from November 2014 until May 2015. Following an incubation under environmental conditions for six months, each sample was supplemented with 10 mL of sterile dH_2_O, and serial dilutions were prepared to measure the culturable count. The log_10_ decline was calculated for each isolate. An analysis of variance (ANOVA) was performed to examine the significance of differences in log decline among groups and sample types using R (v.3.2.2) (44).

## Results

### Isolation of *E. coli*

**Fig. 1:**
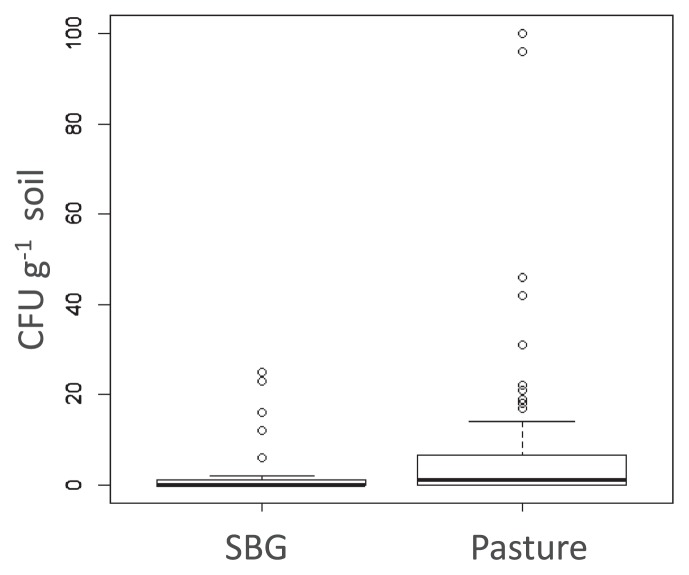
Box and whisker plot showing the culturable population density of *E. coli* in soil before grazing (SBG) and in pasture at the time of grazing (*p*=0.084).

We isolated *E. coli* from pasture soil (120 isolates), run-off (163 isolates), and fresh bovine feces (35 isolates) while cattle were grazing. *E. coli* was also isolated from the same pasture soil before cattle were introduced for the summer, with 45 SBG isolates. SBG samples contained between 0 and 25 CFU g^−1^ of soil, while most pasture samples (soil after grazing) tested positive for *E. coli*, with up to 100 CFU g^−1^ of soil (Fig. 1). While the average culturable count in SBG samples was lower than that during grazing, it was not significant according to the Welch two sample *t*-test (*p*=0.084). Since cattle were removed during the previous summer and re-introduced only after the SBG samples had been collected, these results indicated that at least some strains maintained populations in soil through the previous fall, winter, and spring. It was not possible to control the access of birds or small animals such as rodents to the pasture. Therefore, some *E. coli* may have been deposited by small mammals or birds entering the pasture before or during the sampling period, adding to the diversity obtained.

### Phylogenetic data analysis

**Fig. 2:**
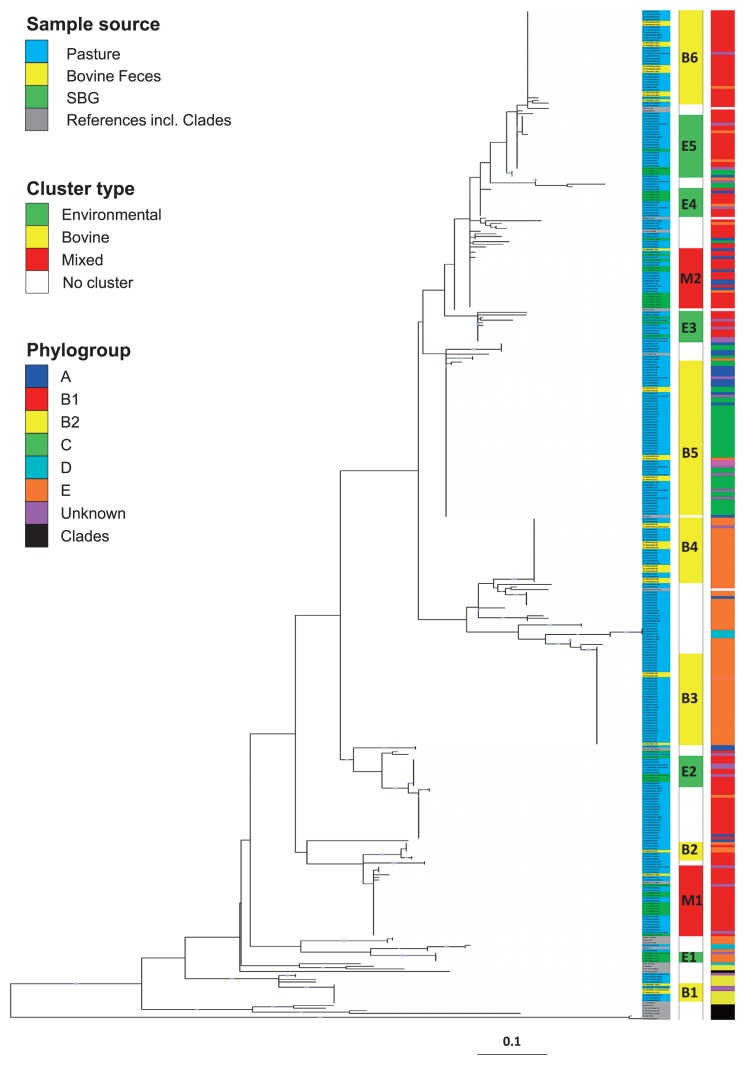
Phylogenetic analysis of concatenated *uidA* and *mutS* gene sequences of *E. coli* isolates, reference strains, and cryptic species of *E. coli* (54). Sequences were aligned using ClustalW and manually trimmed using Se-Al. The best Model: Maximum Likelihood analysis with GTR and G+I was performed using the program MEGA 6. Numbers represent the branch support of 1,000 bootstrap replicates. The phylogenetic tree was color coded and visualized using the Interactive Tree of Life. Isolates are color coded based on their sources (left panel), cluster type (center panel), and phylogroups (right panel). Grey circles on branches indicate a bootstrap value of >80% (1,000 bootstraps).

A phylogenetic analysis of the concatenated *mutS* and *uidA* sequences of all isolates (363 isolates), 25 human pathogen and commensal reference strains (36), and representatives of the cryptic species Clades I, III, IV, and V (54) exhibited multiple distinct clusters (Fig. 2). These clusters were classified into three groups based on the origin of isolates; (i) isolates from all sample types, except bovine feces, *i.e.*, SBG and pasture; designated Environmental, (ii) isolates from all sample types, except SBG, *i.e.*, feces and pasture; designated Bovine, and (iii) groups containing all three sample types; designated Mixed. Only two of the mixed clusters were obtained, and these mostly contained SBG and pasture isolates, and only three bovine isolates. These three bovine isolates may be adapted to the soil and gut environments. Five clusters of isolates fell into the environmental class. Six well-separated bovine clusters were observed, two clusters with strong bootstrap support (Bov-3 and Bov-4 with 100). Most of the reference strains clustered separately from our isolates, indicating different *E. coli* from the current reference strains (36). None of our isolates clustered with any of the cryptic species.

### Phylogroup distribution

**Fig. 3:**
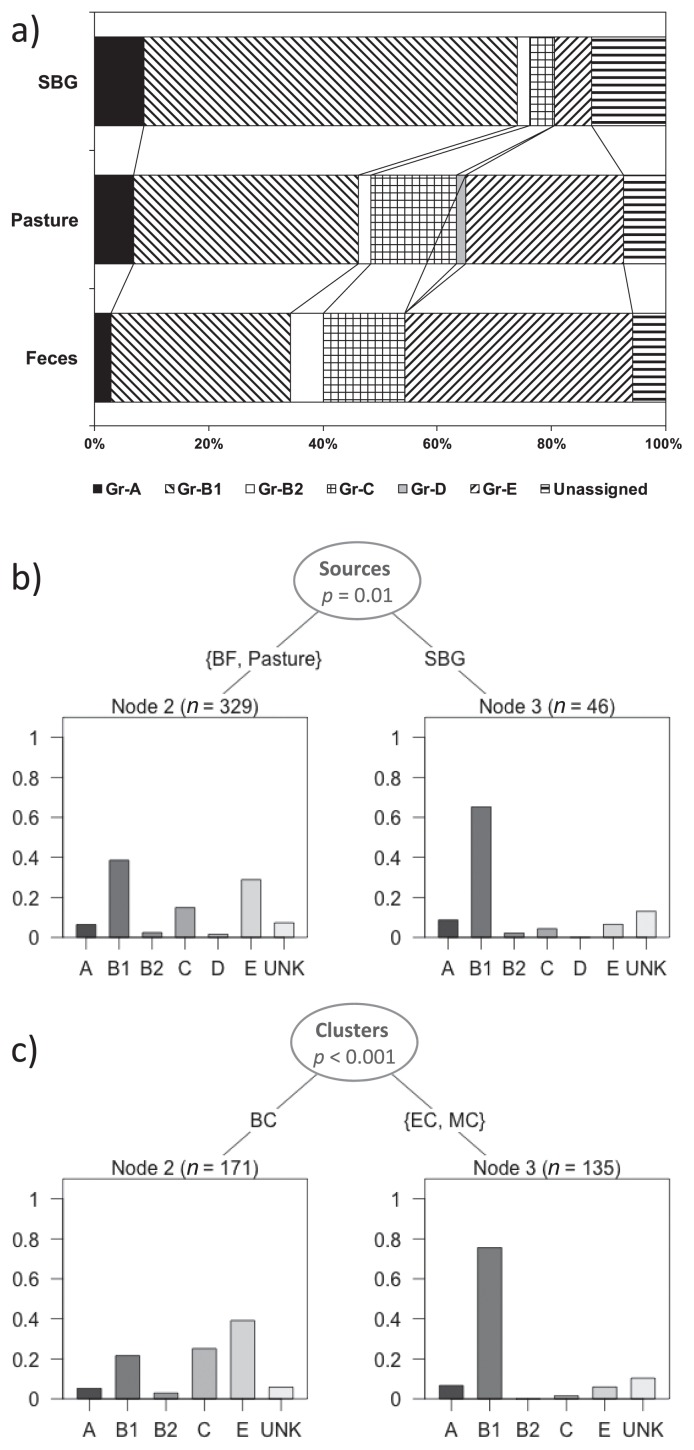
Phylogroup distribution of isolates across sample and cluster types. Distribution of phylogroups of isolates across soil before grazing (SBG), pasture soil while grazing, and bovine feces (a). Phylogrouping was performed according to the scheme of Clermont *et al.* (16). Regression tree showing the difference in the distribution of phylogenetic groups among sources (b) and clusters (c). The X axis denotes phylogroups and the Y-axis represents the proportion of isolates. BF-bovine feces, Past-pasture, SBG-soil before grazing, BC-bovine cluster, EC-environmental cluster, MC-mixed cluster.

Isolates were assigned to six out of seven possible phylogroups: A, B1, B2, C, D, and E. Phylogroup distribution varied across sample types (Fig. 3a). Some isolates were not allocated to any of these phylogroups, and were termed unknown, whereas none of the isolates were assigned to group-F or Clade I. Phylogroup distribution across all isolates showed an overall predominance of B1 and E. SBG samples contained a higher percentage of B1 (66%) than bovine fecal (32%) or pasture samples (40%), indicating that some B1 strains maintained populations in soil slightly more effectively than their counterparts. Bovine fecal isolates displayed a higher percentage of E (40%) than SBG (7%) or pasture samples (28%), suggesting that phylogroup E was primarily bovine-associated and less able to maintain populations in pasture soil. Bovine clusters Bov-3 and Bov-4 were comprised almost exclusively of phylogroup E (Fig. 2). There were very few phylogroup A isolates in feces and soil samples, but the distribution pattern was similar to B1, indicating that A is somewhat more environmental than bovine-associated, but not particularly competitive in either environment. Bov-5 cluster was comprised of mostly phylogroup C, while cluster Bov-6 was primarily phylogroup B1. The distribution of the C-phylogroup was similar to E, suggesting that the source of C in soil is mostly from cattle feces. The distribution of unknown isolates was similar to phylogroup B1.

A multinomial log linear regression analysis of phylogroup distribution based on source types (Fig. 3b) suggested similarity between bovine fecal and pasture communities, while fecal and pasture communities were different from SBG (*p*=0.01), indicating that SBG populations were not from cattle. A cluster-wise comparison of phylogroup distribution revealed that environmental and mixed clusters were similar (Fig. 3c), indicating that the three bovine isolates in mixed clusters were unique, displaying fitness in the bovine colon and pasture. Environmental and mixed cluster compositions differed from bovine cluster populations (*p*<0.001).

### Curli and biofilm formation

**Fig. 4:**
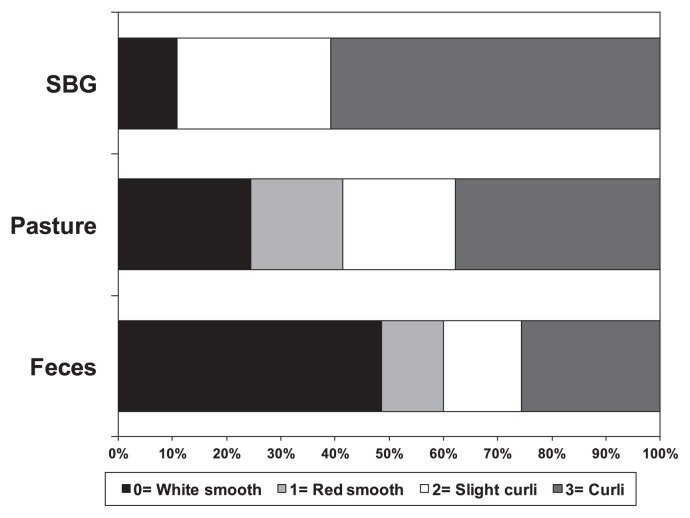
Distribution of RDAR groups among sample sources.

In order to characterize phenotypes potentially associated with long-term survival in soil, isolates were compared for curli formation. RDAR phenotyping showed that 38% of the isolates were intense curli formers. SBG isolates had the greatest proportion of curli formers, whereas pasture isolates (38%) fell between bovine feces (26%) and SBG (61%) (Fig. 4). A chi-squared test of the distribution of RDAR by isolate sources yielded *p*=1.801×10^−11^. This indicated that curli formation is associated with the soil fitness of some, but not all *E. coli*. No clear association was found between curli formation and phylogroup (data not shown), whereas most of phylogroup B1 formed curli.

The degree of biofilm formation varied widely among isolates (Fig. S1), although all isolates formed more sparse biofilms than *P. aeruginosa* PAO (OD=1.36). Many isolates formed more biofilms in LB media than SESOM; however, the reverse was observed for a small number, indicating variations in biofilm formation across the strains. Most SBG isolates formed sparse biofilms, indicating that persistence in soil was not associated with biofilm formation. There was no correlation between curli and biofilm formation in either medium (data not shown), in contrast to previous findings on isolates from a human origin (1). There was also no correlation between phylogroup and biofilm formation (data not shown).

### Winter survival of selected isolates

**Fig. 5:**
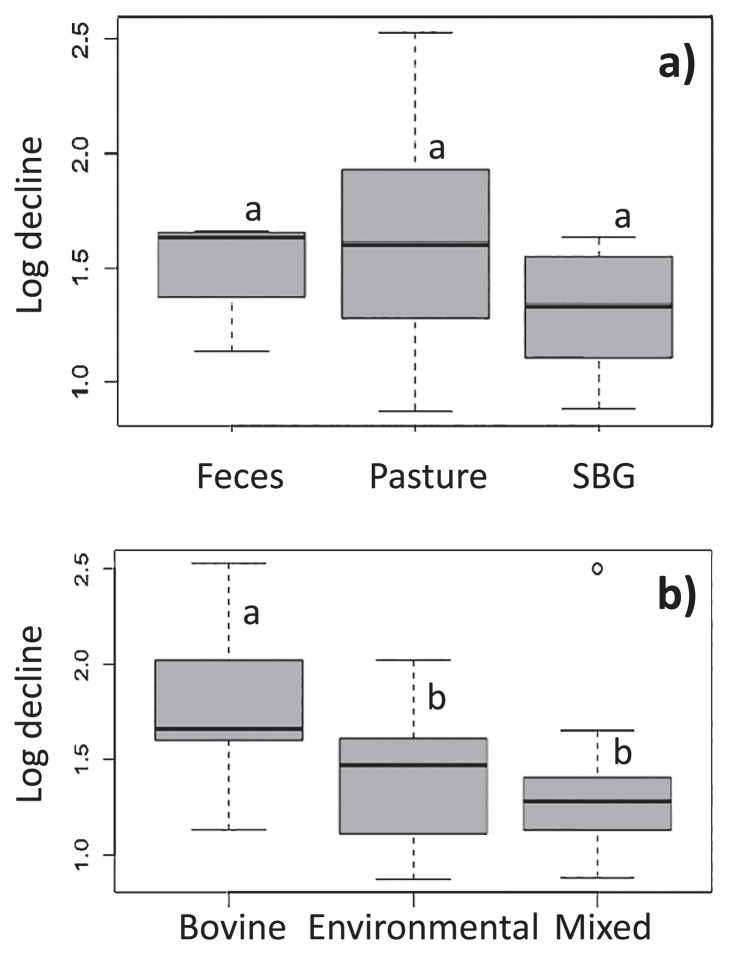
Box and whisker plots showing log_10_ decline in *E. coli* isolates grouped by sample source (a) and clusters (b). Letters denote a significant difference as measured by ANOVA.

In order to study the long-term fate of various isolates in pasture soil, strains selected from all three sample types and clusters were incubated in soil and kept outdoors from November until May. All 45 isolates were recovered after the winter, with the highest population decline being log_10_ 2.5 (Fig. 5a, b). SBG isolates had the lowest average decline; however, due to the few isolates with higher decline, the SBG group was not significantly better than the pasture and fecal isolates (Fig. 5a). Mixed cluster strains demonstrated a significantly greater degree of survival than the bovine cluster (Fig. 5b). Although not significant, environmental cluster strains showed a lower mean decline than bovine strains. This result suggested that mixed and environmental cluster strains had a higher propensity to survive the winter in soil than bovine cluster isolates (*p*<0.05). Although the difference in the log decline was small, it did suggest a difference in fitness that may lead to a shift in the predominance of strains over time. Since sterile soil was used, this experiment was not performed in the presence of competitors; therefore, a greater decline may occur under natural conditions.

## Discussion

We herein attempted to clarify whether *E. coli* from bovine feces differed phenotypically and genotypically from isolates maintaining a population in pasture soil over winter, indicating niche-partitioning. *E. coli* strains that survived in pasture soil through the extreme South Dakota winter displayed a different genotype from bovine fecal isolates, while only a few of the bovine derived genotypes were isolated from pasture after the winter. These results indicated that the niche partitioning of *E. coli* occurs between cattle and their pasture.

The phylogenetic analysis of concatenated *uidA* and *mutS* sequences showed diverse groups of isolates separated into different, well-supported clusters (Fig. 2). It is unlikely that all isolates in one cluster were clonal because multiple samples were taken from across a 12.14-ha surface area populated with multiple cattle. Most of the reference strains used in this study clustered separately from our isolates, but were of human origin, and most of them were pathogenic or related to a clinical condition (36). None of these reference strains clustered in environmental clusters (Fig. 2). Some strains collected before grazing clustered separately from any bovine-associated strains. As shown in previous studies, strains isolated from different aqueous and soil habitats showed a high genetic diversity (8, 20, 25, 34). A large number of soil isolates clustered with bovine isolates, but with no SBG isolates in these clusters, suggesting that they were introduced recently to the pasture through feces. Mixed clusters contained only one and two bovine isolates, indicating that select strains thrive in multiple niches, *i.e.* the bovine gastro-intestinal tract as well as pasture soil under varying weather conditions. Five small clusters contained only SBG and pasture isolates, suggesting these *E. coli* strains had the ability to survive the winter months through freeze-thaw cycles and subsequently grow in the summer months. Since these isolates may be environmentally adapted or naturalized (27), we designated them environmental. *E. coli* populations detected in a number of environments such as soil and water in tropical, temperate, or alpine climates have been designated as naturalized or environmental (4, 7, 12, 27, 50). Approximately 12% of our isolates fell under the environmental category and, thus, may represent multiple naturalized populations adapted to niches in pasture.

*E. coli* is considered to be a highly versatile and diverse species equipped with the ability to survive in many different habitats that are potentially stressful to other strains of the species. The genome flexibility of *E. coli* plays a key role in its metabolic and phenotypic diversity, increasing the competitiveness and fitness of individual variants in specific niches (36). Several groups of phylogenetically distinct *E. coli* found only in the environment were recently grouped into four “Clades”, viz. I, III, IV, and V (39). These were designated by Luo *et al.* (39) as the “Environmental *E. coli*”, implying only cryptic clades are environmental in nature. Walk *et al.* (54) reported that the members of Clades I, III, IV, and V were found in higher abundance in environmental samples than in human isolates. We did not obtain any of these Clade isolates as per the *uidA* and *mutS* sequence analysis or by phylogrouping. Since all 15 clade strains evaluated showed the ability to grow on MLGA, were β-galactosidase-positive, and more than half were also β-glucuronidase-positive, our isolation protocol should have yielded Clade isolates if dominant in pasture samples. Members of Clusters Env 1–5 are possible environmental *sensu stricto E. coli*. In contrast to our results, Clades I, III, IV, and V occur in association with backyard poultry (5).

Most studies have used the initial phylogrouping protocol described by Clermont *et al.* (15). Phylogroups A and B1 are widely viewed as primarily environmental, whereas B2 and D are of mammalian origin (19, 22). The protocol was recently refined to yield eight phylogroups viz. A, B1, B2, C, D, E, F, and Clade I (16). Phylogroup C previously fell within A, and groups E and F fell within D. Phylogroup D was previously viewed as a pathogen group, and the newer group E emanating from it also contains pathogenic strains including enterohemorrhagic *E. coli* such as O157:H7 (36). We obtained many group E isolates, predominantly from bovine feces and recently grazed pasture, consistent with the mammalian association of old phylogroup D. Phylogroup C isolates were primarily from bovine feces and pasture, with only a small proportion from SBG. A small number of Phylogroup B2 isolates were obtained from bovine feces, pasture, or SBG, although B2 is mostly associated with humans and pigs (14). B1 was the most numerous group in pasture soil, both before and after grazing. These have been reported to survive in the environment, and possibly become naturalized in fresh water beaches (53) and estuarine microcosms (3). The difference in the distribution of *E. coli* subpopulations (phylogroups) among various ecological sources is also influenced by fecal deposition by small animals (2).

The RDAR morphotype is associated with the multicellular growth of and biofilm formation by *E. coli* for survival under harsh conditions (55). *E. coli* biofilm formation is associated with the expression of curli and extracellular polymeric substances such as adhesin, amyloid-forming protein, and exopolysaccharide (31, 45, 52). We did not observe a correlation between the phylogroup and RDAR morphotype, or between the RDAR morphotype and biofilm formation, indicating that neither phenotype is conserved phylogenetically across bovine and pasture—associated *E. coli*. Phylogroup B1 was the notable exception with a high proportion of curli-forming isolates. B1 phylogroup isolates from various animals, humans, and water had a markedly higher RDAR positive rate than the other phylogroups (57).

All *E. coli* isolates evaluated showed population survival in soil through the winter. However, the log decline was significantly different between members of the bovine versus mixed clusters. Since the mixed clusters contained only one and two bovine isolates, it is possible that these bovine isolates displayed fitness for the soil and GI tract niches. The overall log decline was not very high, and this may have been because there was no competition with other microorganisms or predators. A previous study reported that *E. coli* K12 viability declined at a higher rate in non-sterile water, soil, and sea-water than under sterile conditions (6). *E. coli* O157–H7 in a pure culture maintained its population until day 30, and there was only one log_10_ decline after day 179. Naturalized *E. coli* isolated from soil survived and grew better in sterile than in non-sterile soil, indicating that the presence of indigenous microbes negatively affected the growth of *E. coli* (30).

## Conclusion

We demonstrated the occurrence of diverse groups of *sensu stricto E. coli* in a cattle pasture, some clustering with SBG, but not cattle isolates, indicating niche partitioning. The strains of these clusters were better able to survive winter freezing in soil. A marked variation in the distribution of phylogroups also described genetic diversity among isolates. Our results add further support to the existence of environmental *sensu stricto* (non-cryptic species) *E. coli* that have become naturalized in soil and forms a reservoir of populations in the environment. Further studies are required in order to characterize the biology of these environmental *E. coli* isolates, including their adaptive abilities and pathogenicity.

## Supplementary Material
