# Supplementary material for: Distribution of Diverse Escherichia coli between Cattle and Pasture
Source: Microbes Environ. 2017 Sep 27;32(3):226–33. doi: 10.1264/jsme2.ME17030 (PMC5606692; doi:10.1264/jsme2.ME17030)
Supplement: Supplementary file 1 [file 32_226_s1.pdf]

Table S1: Primers used for determining the *uidA* and *mutS* sequences, and for phylogrouping.

Primer	Sequence (5' – 3')	Size	Reference
uidA	CATTACGGCAAAGTGTGGGTCAAT (F)<br>TCAGCGTAAGGGTAATGCGAGGTA (R)	658 bp*	(Walk et al., 2009)
mutS	GGCCTATACCCTGAACTACA (F)<br>GCATAAAGGCAATGGTGTC (R)	596 bp	(Walk et al., 2009)
chuA	ATGGTACCGGACGAACCAAC (F)<br>TGCCGCCAGTACCAAAGACA (R)	288 bp	(Clermont et al., 2013)
yjaA	CAAACGTGAAGTGTCAGGAG (F)<br>AATGCGTTCCTCAACCTGTG (R)	211 bp	(Clermont et al., 2013)
TspE4	CACTATTCGTAAGGTCATCC (F)<br>AGTTTATCGCTGCGGGTCGC (R)	152 bp	(Clermont et al., 2013)
arpA	AACGCTATTCGCCAGCTTGC (F)<br>TCTCCCCATACCGTACGCTA (R)	400 bp	(Clermont et al., 2013)
Group E ArpAgpE	GATTCCATCTTGTCAAAATATGCC (F)<br>GAAAAGAAAAAGAATTCCCAAGAG (R)	301 bp	(Clermont et al., 2013)
Group C rpAgpC	AGTTTTATGCCCAGTGCGAG (F)<br>TCTGCGCCGGTCACGCCC (R)	219 bp	(Clermont et al., 2013)

* bp = base pair

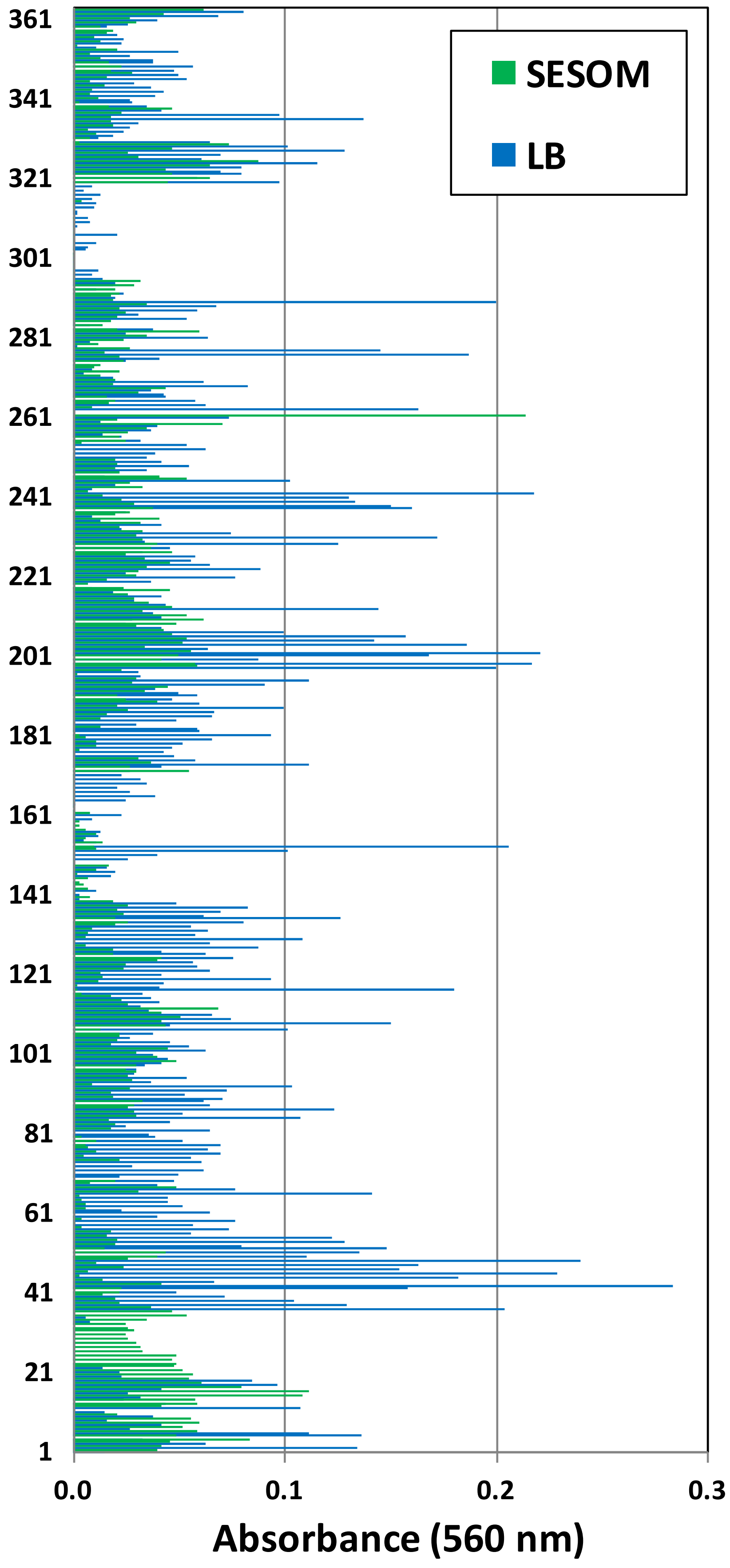

Fig. S1 Distribution of biofilm formationSESOM media. Data represent the average of eight experiments.

Gitanjali NandaKafle
